# Crystal structure and Hirshfeld surface analysis of the ortho­rhom­bic polymorph of a Zn^II^ complex with 3,5-di­nitro­benzoic acid and ethyl­enedi­amine

**DOI:** 10.1107/S2056989020007938

**Published:** 2020-06-19

**Authors:** Avazbek Ibragimov, Jamshid Ashurov, Aziz Dusmatov, Aziz Ibragimov

**Affiliations:** a Institute of Bioorganic Chemistry, Academy of Sciences of Uzbekistan, M. Ulugbek Str., 83, Tashkent, 700125, Uzbekistan; b Agency on Development of the Pharmaceutical Industry, Ch. Aytmatov Str., 1a, Tashkent, 10008, Uzbekistan; c Institute of Total and Inorganic Chemistry, Academy of Sciences of Uzbekistan, M. Ulugbek Str., 77a, Tashkent, 700170, Uzbekistan

**Keywords:** crystal structure, 3,5-di­nitro­benzoic acid, ethyl­enedi­amine, Hirshfeld surface analysis, hydrogen bonding

## Abstract

During systematic investigations of the biological action enhancement of well known compounds, a new metal complex, namely, bis­(3,5-di­nitro­benzoato)(ethane-1,2-di­amine)zinc(II), [Zn(C_7_H_3_N_2_O_6_)_2_(C_2_H_8_N_2_)], was synthesized and the structure of its ortho­rhom­bic form has determined. The synthesis and crystal structure of the monoclinic polymorph has previously been reported [Ibragimov *et al.* (2020 ▸). *Rep. Uzb. Acad. Sci.* **1**, 45–50]. The zinc ion has a distorted tetra­­hedral environment formed by two monodentate 3,5-di­nitro­benzoato anions and chelating ethyl­enedi­amine mol­ecule. In the crystal, the complex mol­ecules are linked by N—H⋯O and C—H⋯O hydrogen bonds into a two-dimensional network parallel to the *ac* plane. The Hirshfeld surface analysis indicates that the most important contributions to the crystal packing are from H⋯O/O⋯H (43.4%) and O⋯C/C⋯O (17.7%) inter­actions.

## Abstract 2

During systematic investigations of the biological action enhancement of well known compounds, a new metal complex, (ethane-1,2-di­amine)­bis­(3,5-di­nitro­benzoato)zinc(II), was synthesized and the structure of its ortho­rhom­bic form has determined. The zinc ion has a distorted tetra­hedral environment formed by two monodentate 3,5-di­nitro­benzoato anions and chelating ethyl­enedi­amine mol­ecule.

## Chemical context   

The benzoic acid derivative 3,5-di­nitro­benzoic acid (DNBA) is an important corrosion inhibitor that is also applied in photography (Elks & Ganellin, 1990 ▸). This aromatic compound is used by chemists in the fluoro­metric analysis of creatinine (Lewinska *et al.*, 2018 ▸; Chandrasekaran *et al.*, 2013 ▸). It demonstrates a weak anti­microbial activity against bacteria and yeasts with a minimum inhibitory concentration (MIC) of 3 mmol L^−1^, but shows moderate biological action with respect to the filamentous fungi *M. gypseum* with IC50 = 2.1 mmol L^−1^ (Vaskova *et al.*, 2009 ▸).

Ethyl­enedi­amine (En) is widely used in the chemical industry. It is a well-known bidentate chelating ligand that donates lone pairs of electrons of two nitro­gen atoms (Matsushita & Taira, 1999 ▸). En is not itself biologically active against different strains of microoraganisms, but its Co^III^ complex demonstrates a strong anti­fungal action relative to a broad spectrum of *Candida* species (Turecka *et al.*, 2018 ▸).

DNBA is poorly water soluble; its solubility is only 1.35 g L^−1^ at 25°C. In order to enhance its water solubility and anti­microbial activity, we tested some of the presently known approaches (Jain *et al.*, 2015 ▸). More promising is a preparation of organic salts of DNBA and En as well as mixed-ligand complexes based on them. Such an approach has been applied for the biopharmaceutical optimization of 4-nitro­benzoic acid (Ibragimov *et al.*, 2017 ▸) and 4-amino­benzoic acid (Ibragimov *et al.*, 2016 ▸) yielding impressive results.

However, an analysis of the Cambridge Structural Database (CSD Version 5.41, update of November 2019; Groom *et al.*, 2016 ▸) attests that organic salts based on DNBA and En have already been obtained as ethyl­endi­ammonium bis­(3,5-di­nitro­benzoate) (refcode VUJXIH; Nethaji *et al.*, 1992 ▸) and ethyl­endi­ammonium bis­(3,5-di­nitro­benzoate) bis­(3,5-di­nitro­benzoic acid) (FONCER; Jones *et al.*, 2005 ▸). Therefore, we synthesized two polymorphic forms of the zinc mixed-ligand complex. The synthesis and crystal structure of the monoclinic polymorph has been published recently (Ibragimov *et al.*, 2020 ▸), and the present paper is devoted to an ortho­rhom­bic polymorph that crystallizes in space group *Pbca*.
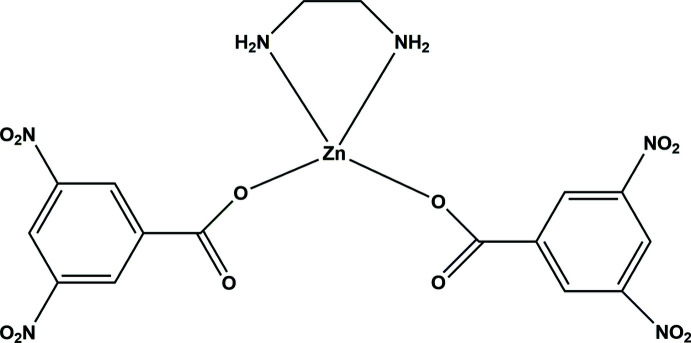


## Structural commentary   

**Figure 1:**
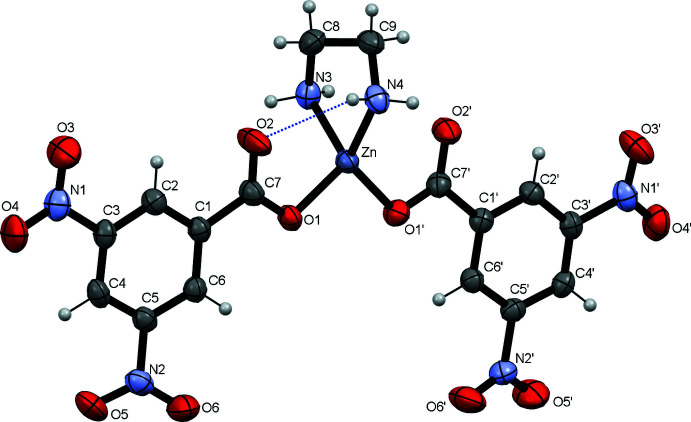
Mol­ecular structure of [Zn(DNBA)_2_(En)] with the atom-numbering scheme. Displacement ellipsoids are drawn at the 50% probability level.

Two DNBA anions coordinate the Zn^II^ ion in a monodentate mode *via* the oxygen atoms of the carboxyl­ate groups. As is usual, the En mol­ecule acts as a chelating ligand through the two nitro­gen atoms (Fig. 1 ▸). The coordination tetra­hedron is distorted because of the Zn1⋯O2 and Zn1⋯O2′ inter­actions, the angles N3—Zn1—N4 [87.09 (7)°] and O1—Zn1—O1′ [101.82 (5)°] being less than the idealized tetra­hedral values. The least-squares planes through the nitro groups are almost parallel to the planes of the aromatic rings. The nitro group N2′O5′O6′ subtends the largest dihedral angle to the attached aromatic ring [16.65 (11)°]. The conformation of the complex mol­ecule is fixed due to the intra­molecular N4—H4*A*⋯O2 hydrogen bond, which closes a six-membered ring with graph-set notation *S*(6) (Etter, 1990 ▸).

## Supra­molecular features   

**Figure 2:**
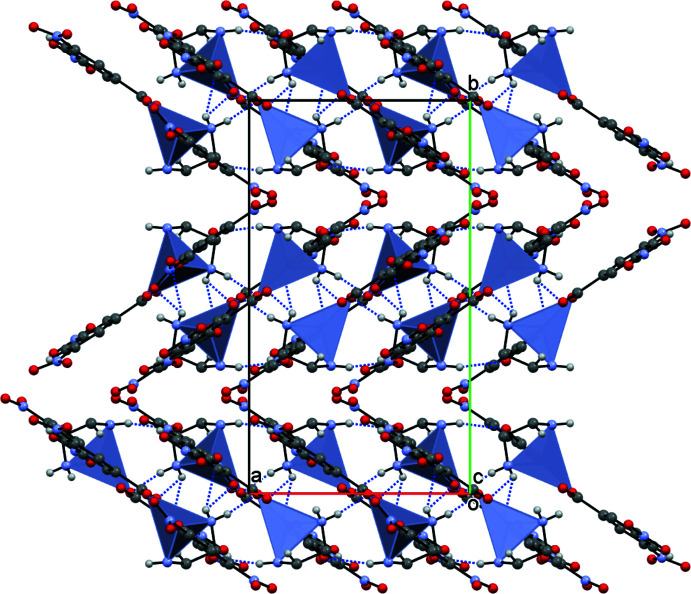
A packing diagram for [Zn(DNBA)_2_(En)] showing the two-dimensional networks parallel to (010). For clarity, H atoms not involved in hydrogen bonding are omitted.

In the crystal, complex mol­ecules are linked by three relatively weak hydrogen bonds of the N—H⋯O type and two bonds of C—H⋯O type (Table 1 ▸). The N3—H3*A*⋯O2′, N4—H4*A*⋯O5′ and N4—H4*B*⋯O1 hydrogen bonds link the complex mol­ecules into a two-dimensional network parallel to the *ac* plane. Weak C6′—H6′⋯O6′ and C8—H8*B*⋯O3 hydrogen bonds strengthen the association of the complex mol­ecules into this network (Fig. 2 ▸). Thus, only the H3*B* hydrogen on the N3 atom is without an acceptor and five oxygen atoms O1′, O3′, O4′, O4 and O5 do not participate in hydrogen bonding.

## Hirshfeld surface analysis   

**Figure 3:**
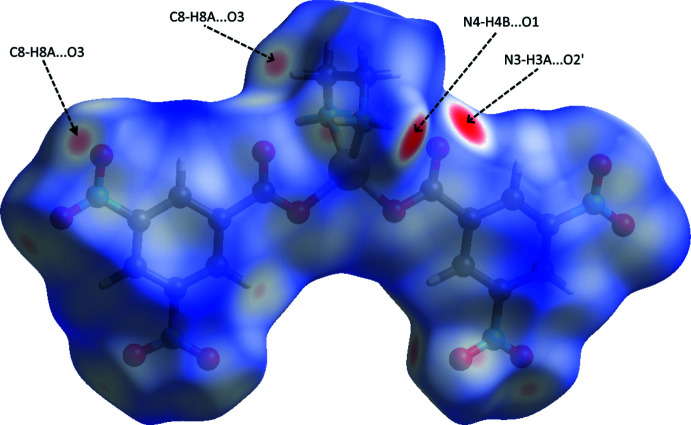
View of the three-dimensional Hirshfeld surface of the title compound plotted over *d*_norm_ in the range −0.4180 to 1.3344 a.u.

**Figure 4:**
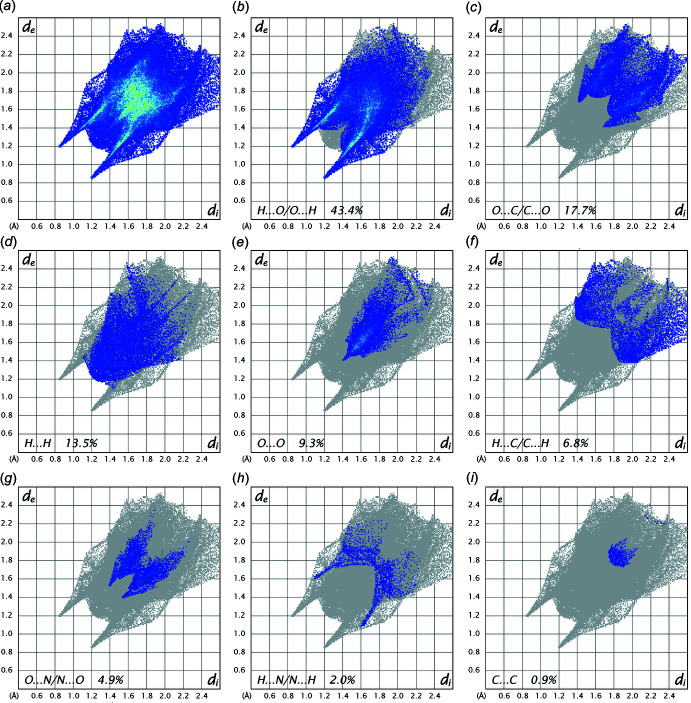
Full two-dimensional fingerprint plots for the title compound, showing all inter­actions (*a*), and delineated into (*b*) H⋯O/O⋯H, (*c*) O⋯C/C⋯O, (*d*) H⋯H, (*e*) O⋯O, (*f*) H⋯C/C⋯H, (*g*) O⋯N/N⋯O, (*h*) H⋯N/N⋯H and (*i*) C⋯C inter­actions. The *d*_i_ and *d*_e_ values are the closest inter­nal and external distances (in Å) from a given point on the Hirshfeld surface.

In order to visualize the inter­molecular inter­actions in the crystals of the title compound, a Hirshfeld surface analysis was carried out using *Crystal Explorer 17.5* (Turner *et al.*, 2017 ▸). The Hirshfeld surface mapped over *d*_norm_ (Fig. 3 ▸) shows the expected bright-red spots near atoms O1, O2, O2, O3, O5′, O6, H3*A*, H4*A*, H4′, H4*B* and H8*A* involved in the N—H⋯O and C—H⋯O hydrogen-bonding inter­actions described above. Fingerprint plots (Fig. 4 ▸) reveal that while H⋯O/O⋯H inter­actions make the greatest contributions to the surface contacts, as would be expected for a mol­ecule with such a predominance of oxygen atoms, O⋯C/C⋯O, H⋯H and O⋯O contacts are also substantial (Table 2 ▸), while H⋯C/C⋯H, O⋯N/N⋯O, H⋯N/N⋯H, C⋯C, N⋯C/C⋯N and N⋯N contacts are less significant.

## Database survey   

A search of the Cambridge Structural Database (CSD Version 5.41, update of November 2019; Groom *et al.*, 2016 ▸) found 277 metal complexes involving DNBA. Among them, 29 hits are zinc complexes, of which 14 have the coordination number four. In all of these complexes, two DNBA anions are coordinated in a monodentate fashion and only in structures JOHYEN (Torres *et al.*, 2019 ▸) and VIQFAE (Dey *et al.*, 2013 ▸) is the coordination of Zn^II^ accomplished by chelating ligands: 1,1′-methyl­enebis(3,5-dimethyl-1*H*-pyrazole and 2,2′-bipyridyl for JOHYEN and VIQFAE, respectively.

## Synthesis and crystallization   

To an aqueous solution (2.5 ml) of ZnCl_2_ (0,068 g, 0.5 mmol) was slowly added a mixture of ethanol (4 ml), En (60 µL) and DNBA (0.212 g, 1 mmol) under constant stirring. A white crystalline product was obtained at room temperature by slow solvent evaporation after 5 d, yield: 70%. Elemental analysis for C_16_H_14_N_6_O_12_Zn (547.70): calculated C: 35.09, H: 2.58, N:15.34%; found: C: 35.12, H: 2.62, N: 15.41%.

## Refinement   

Crystal data, data collection and structure refinement details are summarized in Table 3 ▸. C-bound hydrogen atoms were placed in calculated positions and refined using the riding-model approximation with *U*_iso_(H) = 1.2*U*_eq_(C), C—H = 0.93 and 0.97 Å for aromatic and methyl­ene hydrogen atoms, respectively. N-bound H atoms were located in a difference-Fourier map and refined with bond-length restraints of 0.89 (1) Å.

## Supplementary Material

Crystal structure: contains datablock(s) I. DOI: 10.1107/S2056989020007938/yk2132sup1.cif

Structure factors: contains datablock(s) I. DOI: 10.1107/S2056989020007938/yk2132Isup2.hkl

CCDC reference: 2009339

Additional supporting information:  crystallographic information; 3D view; checkCIF report

## Figures and Tables

**Table 1:** Hydrogen-bond geometry (Å, °)

*D*—H⋯*A*	*D*—H	H⋯*A*	*D*⋯*A*	*D*—H⋯*A*
C4′—H4′⋯O6^i^	0.93	2.63	3.539 (2)	167
C8—H8*A*⋯O3^ii^	0.97	2.51	3.353 (3)	145
N3—H3*A*⋯O2′^iii^	0.89 (1)	2.19 (1)	3.055 (2)	165 (2)
N4—H4*A*⋯O2	0.89 (1)	2.42 (2)	3.010 (2)	124 (2)
N4—H4*A*⋯O5′^iv^	0.89 (1)	2.58 (2)	3.273 (3)	136 (2)
N4—H4*B*⋯O1^v^	0.88 (1)	2.18 (1)	3.021 (2)	159 (2)

Symmetry codes: (i) 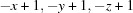; (ii) 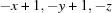; (iii) 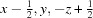; (iv) 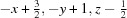; (v) 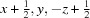.

**Table 2:** Percentage contributions to the Hirshfeld surface for [Zn(DNBA)_2_(En)]

Contacts	Included surface area %
H⋯O/O⋯H	43.4
O⋯C/C⋯O	17.7
H⋯H	13.5
H⋯C/C⋯H	6.8
O⋯N/N⋯O	4.9
H⋯N/N⋯H	2.0
C⋯C	0.9
N⋯C/C⋯N	0.4
N⋯N	0.1

**Table 3:** Experimental details

Crystal data	
Chemical formula	[Zn(C_7_H_3_N_2_O_6_)_2_(C_2_H_8_N_2_)]
*M*_r_	547.70
Crystal system, space group	Orthorhombic, *P* *b* *c* *a*
Temperature (K)	293
*a*, *b*, *c* (Å)	10.26799 (6), 18.26557 (10), 21.67365 (12)
*V* (Å^3^)	4064.91 (4)
*Z*	8
Radiation type	Cu *K*α
μ (mm^−1^)	2.45
Crystal size (mm)	0.42 × 0.3 × 0.18

Data collection	
Diffractometer	Rigaku Oxford Diffraction Xcalibur, Ruby
Absorption correction	Multi-scan (*CrysAlis PRO*; Rigaku OD, 2015 ▸)
*T*_min_, *T*_max_	0.613, 1.000
No. of measured, independent and observed [*I* > 2σ(*I*)] reflections	26085, 4214, 4070
*R*_int_	0.024
(sin θ/λ)_max_ (Å^−1^)	0.629

Refinement	
*R*[*F* ^2^ > 2σ(*F* ^2^)], *wR*(*F* ^2^), *S*	0.032, 0.091, 1.09
No. of reflections	4214
No. of parameters	333
No. of restraints	4
H-atom treatment	H atoms treated by a mixture of independent and constrained refinement
Δρ_max_, Δρ_min_ (e Å^−3^)	0.31, −0.52

Computer programs: *CrysAlis PRO* (Rigaku OD, 2015 ▸), *SHELXT* (Sheldrick, 2015*a* ▸), *SHELXL2018/3* (Sheldrick, 2015*b* ▸), *Mercury* (Macrae *et al.*, 2020 ▸) and *publCIF* (Westrip, 2010 ▸).

## supplementary crystallographic information

### Crystal data

**Table:**

[Zn(C_7_H_3_N_2_O_6_)_2_(C_2_H_8_N_2_)]	*D*_x_ = 1.790 Mg m^−^^3^
*M**_r_* = 547.70	Cu *K*α radiation, λ = 1.54184 Å
Orthorhombic, *P**b**c**a*	Cell parameters from 19707 reflections
*a* = 10.26799 (6) Å	θ = 4.3–76.0°
*b* = 18.26557 (10) Å	µ = 2.44 mm^−^^1^
*c* = 21.67365 (12) Å	*T* = 293 K
*V* = 4064.91 (4) Å^3^	Block, white
*Z* = 8	0.42 × 0.3 × 0.18 mm
*F*(000) = 2224	

### Data collection

**Table:**

Rigaku Oxford Diffraction Xcalibur, Ruby diffractometer	4214 independent reflections
Radiation source: Enhance (Cu) X-ray Source	4070 reflections with *I* > 2σ(*I*)
Graphite monochromator	*R*_int_ = 0.024
Detector resolution: 10.2576 pixels mm^-1^	θ_max_ = 76.0°, θ_min_ = 4.1°
ω scans	*h* = −12→12
Absorption correction: multi-scan (CrysAlisPro; Rigaku OD, 2015)	*k* = −20→22
*T*_min_ = 0.613, *T*_max_ = 1.000	*l* = −20→27
26085 measured reflections	

### Refinement

**Table:**

Refinement on *F*^2^	Hydrogen site location: mixed
Least-squares matrix: full	H atoms treated by a mixture of independent and constrained refinement
*R*[*F*^2^ > 2σ(*F*^2^)] = 0.032	*w* = 1/[σ^2^(*F*_o_^2^) + (0.053*P*)^2^ + 1.5574*P*] where *P* = (*F*_o_^2^ + 2*F*_c_^2^)/3
*wR*(*F*^2^) = 0.091	(Δ/σ)_max_ = 0.002
*S* = 1.09	Δρ_max_ = 0.31 e Å^−^^3^
4214 reflections	Δρ_min_ = −0.52 e Å^−^^3^
333 parameters	Extinction correction: SHELXL-2018/3 (Sheldrick 2018), Fc^*^=kFc[1+0.001xFc^2^λ^3^/sin(2θ)]^-1/4^
4 restraints	Extinction coefficient: 0.00077 (6)

### Special details

**Table:**

Geometry. All esds (except the esd in the dihedral angle between two l.s. planes) are estimated using the full covariance matrix. The cell esds are taken into account individually in the estimation of esds in distances, angles and torsion angles; correlations between esds in cell parameters are only used when they are defined by crystal symmetry. An approximate (isotropic) treatment of cell esds is used for estimating esds involving l.s. planes.

### Fractional atomic coordinates and isotropic or equivalent isotropic displacement parameters (Å^2^)

**Table:**

	*x*	*y*	*z*	*U*_iso_*/*U*_eq_	
Zn1	0.67282 (2)	0.42317 (2)	0.24573 (2)	0.03229 (10)	
O1	0.53739 (12)	0.49815 (7)	0.23272 (6)	0.0371 (3)	
O1'	0.68069 (13)	0.41811 (7)	0.33561 (6)	0.0414 (3)	
O4'	1.02982 (15)	0.26641 (9)	0.58578 (7)	0.0576 (4)	
O2'	0.83739 (14)	0.33654 (10)	0.31937 (6)	0.0544 (4)	
O5	0.02506 (14)	0.68636 (8)	0.22153 (8)	0.0551 (4)	
N3	0.62989 (16)	0.32537 (8)	0.20461 (7)	0.0396 (3)	
N1'	1.02277 (14)	0.27851 (8)	0.53038 (8)	0.0410 (3)	
N4	0.82786 (14)	0.42902 (9)	0.18869 (8)	0.0389 (3)	
O3	0.28325 (19)	0.57939 (10)	−0.01964 (7)	0.0670 (5)	
O6'	0.57039 (17)	0.47877 (10)	0.55318 (8)	0.0695 (5)	
N2'	0.64170 (16)	0.43071 (9)	0.57126 (7)	0.0419 (4)	
O4	0.11575 (17)	0.64392 (10)	0.00591 (7)	0.0634 (4)	
O2	0.57988 (17)	0.48659 (12)	0.13270 (7)	0.0750 (6)	
N1	0.21307 (17)	0.60866 (9)	0.01838 (7)	0.0459 (4)	
N2	0.13183 (16)	0.66326 (8)	0.23606 (8)	0.0425 (4)	
C1	0.39543 (16)	0.55533 (9)	0.16039 (8)	0.0324 (3)	
C2'	0.89150 (16)	0.32663 (9)	0.44488 (8)	0.0336 (3)	
H2'	0.947315	0.302924	0.417622	0.040*	
C5'	0.72941 (16)	0.39536 (9)	0.52664 (7)	0.0333 (3)	
C6'	0.70569 (16)	0.40357 (9)	0.46427 (8)	0.0326 (3)	
H6'	0.636643	0.432089	0.450362	0.039*	
C1'	0.78781 (15)	0.36807 (9)	0.42272 (7)	0.0314 (3)	
C5	0.21151 (16)	0.62756 (9)	0.18838 (8)	0.0345 (3)	
C3'	0.91054 (16)	0.32112 (9)	0.50751 (8)	0.0333 (3)	
C2	0.35964 (17)	0.56172 (9)	0.09885 (8)	0.0350 (3)	
H2	0.409358	0.539865	0.068072	0.042*	
C4	0.17179 (16)	0.63447 (9)	0.12799 (9)	0.0372 (4)	
H4	0.096968	0.660232	0.117358	0.045*	
C7	0.51413 (17)	0.50986 (10)	0.17523 (8)	0.0377 (4)	
C3	0.24905 (17)	0.60106 (9)	0.08403 (8)	0.0354 (3)	
C6	0.32130 (15)	0.58895 (9)	0.20600 (8)	0.0336 (3)	
H6	0.344937	0.585566	0.247323	0.040*	
C4'	0.83052 (15)	0.35433 (9)	0.55020 (8)	0.0335 (3)	
H4'	0.843953	0.349357	0.592423	0.040*	
C7'	0.76869 (16)	0.37360 (10)	0.35383 (7)	0.0354 (3)	
C8	0.7149 (2)	0.32148 (11)	0.14984 (9)	0.0461 (4)	
H8A	0.675173	0.348006	0.115951	0.055*	
H8B	0.725379	0.270824	0.137340	0.055*	
C9	0.84714 (18)	0.35432 (11)	0.16435 (9)	0.0434 (4)	
H9A	0.891841	0.324435	0.194691	0.052*	
H9B	0.899990	0.356017	0.127285	0.052*	
O5'	0.64367 (18)	0.40972 (11)	0.62426 (7)	0.0680 (5)	
O6	0.17650 (17)	0.66780 (11)	0.28763 (8)	0.0723 (5)	
O3'	1.10353 (16)	0.25867 (9)	0.49322 (7)	0.0613 (4)	
H3A	0.5474 (12)	0.3214 (13)	0.1932 (11)	0.053 (6)*	
H3B	0.647 (2)	0.2916 (10)	0.2324 (9)	0.052 (6)*	
H4A	0.804 (2)	0.4583 (12)	0.1581 (9)	0.058 (7)*	
H4B	0.9014 (15)	0.4475 (13)	0.2027 (11)	0.060 (7)*	

### Atomic displacement parameters (Å^2^)

**Table:**

	*U*^11^	*U*^22^	*U*^33^	*U*^12^	*U*^13^	*U*^23^
Zn1	0.03023 (15)	0.03616 (16)	0.03048 (15)	0.00523 (8)	−0.00034 (8)	0.00139 (8)
O1	0.0346 (6)	0.0406 (6)	0.0359 (6)	0.0097 (5)	−0.0028 (5)	0.0036 (5)
O1'	0.0477 (7)	0.0479 (7)	0.0286 (6)	0.0089 (5)	−0.0025 (5)	0.0032 (5)
O4'	0.0614 (9)	0.0618 (9)	0.0495 (8)	0.0088 (7)	−0.0174 (7)	0.0118 (7)
O2'	0.0478 (8)	0.0838 (11)	0.0315 (7)	0.0216 (7)	0.0036 (5)	−0.0027 (7)
O5	0.0417 (7)	0.0530 (8)	0.0707 (10)	0.0174 (6)	0.0058 (7)	0.0022 (7)
N3	0.0381 (8)	0.0375 (8)	0.0432 (8)	−0.0031 (6)	−0.0070 (6)	0.0030 (6)
N1'	0.0356 (7)	0.0349 (7)	0.0524 (9)	−0.0006 (6)	−0.0056 (7)	0.0090 (6)
N4	0.0312 (8)	0.0455 (9)	0.0402 (9)	−0.0006 (6)	0.0017 (6)	0.0066 (6)
O3	0.0748 (11)	0.0892 (13)	0.0370 (8)	0.0189 (9)	0.0014 (8)	0.0096 (7)
O6'	0.0760 (11)	0.0794 (11)	0.0530 (9)	0.0400 (9)	0.0073 (8)	−0.0040 (8)
N2'	0.0421 (8)	0.0484 (9)	0.0351 (8)	0.0024 (7)	0.0012 (6)	−0.0068 (6)
O4	0.0625 (9)	0.0736 (10)	0.0542 (9)	0.0172 (8)	−0.0210 (7)	0.0129 (8)
O2	0.0726 (11)	0.1122 (15)	0.0401 (8)	0.0598 (11)	0.0008 (7)	0.0021 (8)
N1	0.0489 (9)	0.0474 (9)	0.0414 (8)	−0.0008 (7)	−0.0080 (7)	0.0114 (7)
N2	0.0415 (8)	0.0343 (7)	0.0518 (9)	0.0064 (7)	0.0014 (7)	−0.0021 (6)
C1	0.0305 (8)	0.0299 (7)	0.0368 (8)	0.0014 (6)	−0.0025 (6)	0.0030 (6)
C2'	0.0315 (8)	0.0338 (8)	0.0355 (8)	0.0009 (6)	0.0033 (6)	0.0015 (6)
C5'	0.0347 (8)	0.0325 (7)	0.0326 (8)	−0.0031 (6)	0.0013 (6)	−0.0029 (6)
C6'	0.0324 (8)	0.0328 (7)	0.0327 (8)	0.0009 (6)	−0.0011 (6)	0.0019 (6)
C1'	0.0313 (7)	0.0321 (7)	0.0307 (8)	−0.0026 (6)	0.0004 (6)	0.0022 (6)
C5	0.0315 (8)	0.0299 (7)	0.0423 (9)	0.0013 (6)	−0.0002 (7)	−0.0001 (6)
C3'	0.0299 (8)	0.0310 (7)	0.0389 (8)	−0.0025 (6)	−0.0026 (6)	0.0052 (6)
C2	0.0352 (8)	0.0327 (8)	0.0371 (9)	0.0007 (7)	0.0002 (7)	0.0042 (6)
C4	0.0299 (8)	0.0332 (8)	0.0484 (10)	0.0031 (6)	−0.0064 (7)	0.0054 (7)
C7	0.0363 (8)	0.0390 (8)	0.0378 (9)	0.0093 (7)	−0.0025 (7)	0.0039 (7)
C3	0.0344 (8)	0.0350 (8)	0.0369 (8)	−0.0026 (7)	−0.0063 (7)	0.0072 (6)
C6	0.0324 (8)	0.0320 (8)	0.0364 (9)	0.0007 (6)	−0.0042 (6)	0.0014 (6)
C4'	0.0363 (8)	0.0353 (8)	0.0289 (8)	−0.0057 (6)	−0.0035 (6)	0.0020 (6)
C7'	0.0324 (8)	0.0435 (9)	0.0304 (8)	−0.0018 (7)	0.0012 (6)	0.0024 (6)
C8	0.0520 (11)	0.0485 (10)	0.0376 (9)	0.0067 (9)	−0.0072 (8)	−0.0096 (8)
C9	0.0383 (9)	0.0543 (11)	0.0376 (9)	0.0118 (8)	0.0030 (7)	−0.0031 (8)
O5'	0.0750 (11)	0.0951 (13)	0.0339 (8)	0.0224 (10)	0.0123 (7)	0.0028 (8)
O6	0.0726 (12)	0.0943 (13)	0.0500 (10)	0.0323 (9)	−0.0066 (8)	−0.0238 (9)
O3'	0.0454 (8)	0.0677 (10)	0.0709 (10)	0.0218 (7)	0.0111 (7)	0.0211 (8)

### Geometric parameters (Å, º)

**Table:**

Zn1—O1	1.9721 (12)	C1—C2	1.388 (2)
Zn1—O1'	1.9519 (13)	C1—C7	1.509 (2)
Zn1—N3	2.0445 (15)	C1—C6	1.391 (2)
Zn1—N4	2.0184 (15)	C2'—H2'	0.9300
O1—C7	1.287 (2)	C2'—C1'	1.392 (2)
O1'—C7'	1.278 (2)	C2'—C3'	1.375 (2)
O4'—N1'	1.223 (2)	C5'—C6'	1.382 (2)
O2'—C7'	1.230 (2)	C5'—C4'	1.378 (2)
O5—N2	1.216 (2)	C6'—H6'	0.9300
N3—C8	1.475 (3)	C6'—C1'	1.394 (2)
N3—H3A	0.885 (10)	C1'—C7'	1.509 (2)
N3—H3B	0.879 (10)	C5—C4	1.377 (2)
N1'—C3'	1.476 (2)	C5—C6	1.383 (2)
N1'—O3'	1.211 (2)	C3'—C4'	1.378 (2)
N4—C9	1.476 (3)	C2—H2	0.9300
N4—H4A	0.886 (10)	C2—C3	1.382 (2)
N4—H4B	0.881 (10)	C4—H4	0.9300
O3—N1	1.218 (2)	C4—C3	1.382 (3)
O6'—N2'	1.208 (2)	C6—H6	0.9300
N2'—C5'	1.471 (2)	C4'—H4'	0.9300
N2'—O5'	1.211 (2)	C8—H8A	0.9700
O4—N1	1.219 (2)	C8—H8B	0.9700
O2—C7	1.219 (2)	C8—C9	1.517 (3)
N1—C3	1.477 (2)	C9—H9A	0.9700
N2—C5	1.470 (2)	C9—H9B	0.9700
N2—O6	1.211 (2)		
			
O1—Zn1—N3	113.10 (6)	C1'—C6'—H6'	120.8
O1—Zn1—N4	115.58 (6)	C2'—C1'—C6'	119.53 (15)
O1'—Zn1—O1	101.82 (5)	C2'—C1'—C7'	118.50 (14)
O1'—Zn1—N3	113.74 (6)	C6'—C1'—C7'	121.97 (15)
O1'—Zn1—N4	125.53 (6)	C4—C5—N2	117.56 (15)
N4—Zn1—N3	87.09 (7)	C4—C5—C6	123.40 (16)
C7—O1—Zn1	112.64 (11)	C6—C5—N2	119.04 (16)
C7'—O1'—Zn1	111.57 (11)	C2'—C3'—N1'	118.75 (15)
Zn1—N3—H3A	113.6 (16)	C2'—C3'—C4'	123.08 (15)
Zn1—N3—H3B	105.8 (16)	C4'—C3'—N1'	118.17 (15)
C8—N3—Zn1	105.38 (11)	C1—C2—H2	120.5
C8—N3—H3A	109.7 (16)	C3—C2—C1	118.99 (16)
C8—N3—H3B	113.7 (16)	C3—C2—H2	120.5
H3A—N3—H3B	109 (2)	C5—C4—H4	121.8
O4'—N1'—C3'	118.10 (16)	C5—C4—C3	116.43 (15)
O3'—N1'—O4'	123.92 (16)	C3—C4—H4	121.8
O3'—N1'—C3'	117.96 (15)	O1—C7—C1	116.60 (15)
Zn1—N4—H4A	105.7 (16)	O2—C7—O1	124.85 (16)
Zn1—N4—H4B	119.1 (17)	O2—C7—C1	118.55 (16)
C9—N4—Zn1	106.00 (11)	C2—C3—N1	118.59 (16)
C9—N4—H4A	109.1 (17)	C2—C3—C4	122.76 (16)
C9—N4—H4B	111.2 (17)	C4—C3—N1	118.64 (15)
H4A—N4—H4B	105 (2)	C1—C6—H6	120.8
O6'—N2'—C5'	118.47 (16)	C5—C6—C1	118.36 (16)
O6'—N2'—O5'	123.21 (17)	C5—C6—H6	120.8
O5'—N2'—C5'	118.32 (16)	C5'—C4'—H4'	122.0
O3—N1—O4	124.53 (17)	C3'—C4'—C5'	116.09 (15)
O3—N1—C3	117.56 (16)	C3'—C4'—H4'	122.0
O4—N1—C3	117.92 (17)	O1'—C7'—C1'	116.11 (14)
O5—N2—C5	118.25 (16)	O2'—C7'—O1'	124.60 (16)
O6—N2—O5	123.82 (17)	O2'—C7'—C1'	119.29 (15)
O6—N2—C5	117.92 (16)	N3—C8—H8A	109.6
C2—C1—C7	117.68 (15)	N3—C8—H8B	109.6
C2—C1—C6	120.06 (15)	N3—C8—C9	110.10 (14)
C6—C1—C7	122.24 (15)	H8A—C8—H8B	108.2
C1'—C2'—H2'	120.4	C9—C8—H8A	109.6
C3'—C2'—H2'	120.4	C9—C8—H8B	109.6
C3'—C2'—C1'	119.28 (15)	N4—C9—C8	108.63 (14)
C6'—C5'—N2'	119.19 (15)	N4—C9—H9A	110.0
C4'—C5'—N2'	117.16 (15)	N4—C9—H9B	110.0
C4'—C5'—C6'	123.65 (15)	C8—C9—H9A	110.0
C5'—C6'—H6'	120.8	C8—C9—H9B	110.0
C5'—C6'—C1'	118.36 (15)	H9A—C9—H9B	108.3
			
Zn1—O1—C7—O2	−10.6 (3)	C5'—C6'—C1'—C7'	179.61 (15)
Zn1—O1—C7—C1	168.80 (11)	C6'—C5'—C4'—C3'	0.5 (2)
Zn1—O1'—C7'—O2'	−2.2 (2)	C6'—C1'—C7'—O1'	6.4 (2)
Zn1—O1'—C7'—C1'	177.15 (11)	C6'—C1'—C7'—O2'	−174.22 (17)
Zn1—N3—C8—C9	37.33 (17)	C1'—C2'—C3'—N1'	−178.51 (14)
Zn1—N4—C9—C8	40.97 (17)	C1'—C2'—C3'—C4'	0.7 (3)
O4'—N1'—C3'—C2'	−171.17 (16)	C5—C4—C3—N1	−178.17 (15)
O4'—N1'—C3'—C4'	9.6 (2)	C5—C4—C3—C2	0.9 (3)
O5—N2—C5—C4	11.9 (2)	C3'—C2'—C1'—C6'	0.4 (2)
O5—N2—C5—C6	−167.92 (17)	C3'—C2'—C1'—C7'	179.87 (15)
N3—C8—C9—N4	−54.1 (2)	C2—C1—C7—O1	−172.74 (16)
N1'—C3'—C4'—C5'	178.08 (14)	C2—C1—C7—O2	6.7 (3)
O3—N1—C3—C2	0.2 (3)	C2—C1—C6—C5	0.8 (2)
O3—N1—C3—C4	179.28 (18)	C4—C5—C6—C1	0.0 (3)
O6'—N2'—C5'—C6'	−16.6 (2)	C7—C1—C2—C3	177.96 (15)
O6'—N2'—C5'—C4'	164.53 (18)	C7—C1—C6—C5	−177.85 (15)
N2'—C5'—C6'—C1'	−178.32 (15)	C6—C1—C2—C3	−0.8 (2)
N2'—C5'—C4'—C3'	179.34 (14)	C6—C1—C7—O1	6.0 (2)
O4—N1—C3—C2	−179.51 (18)	C6—C1—C7—O2	−174.6 (2)
O4—N1—C3—C4	−0.4 (3)	C6—C5—C4—C3	−0.8 (3)
N2—C5—C4—C3	179.36 (15)	C4'—C5'—C6'—C1'	0.5 (2)
N2—C5—C6—C1	179.82 (15)	O5'—N2'—C5'—C6'	162.72 (18)
C1—C2—C3—N1	178.93 (15)	O5'—N2'—C5'—C4'	−16.1 (3)
C1—C2—C3—C4	−0.1 (3)	O6—N2—C5—C4	−168.19 (18)
C2'—C1'—C7'—O1'	−173.09 (15)	O6—N2—C5—C6	12.0 (3)
C2'—C1'—C7'—O2'	6.3 (2)	O3'—N1'—C3'—C2'	10.0 (2)
C2'—C3'—C4'—C5'	−1.1 (2)	O3'—N1'—C3'—C4'	−169.18 (17)
C5'—C6'—C1'—C2'	−0.9 (2)		

### Hydrogen-bond geometry (Å, º)

**Table:**

*D*—H···*A*	*D*—H	H···*A*	*D*···*A*	*D*—H···*A*
C4′—H4′···O6^i^	0.93	2.63	3.539 (2)	167
C8—H8*A*···O3^ii^	0.97	2.51	3.353 (3)	145
N3—H3*A*···O2′^iii^	0.89 (1)	2.19 (1)	3.055 (2)	165 (2)
N4—H4*A*···O2	0.89 (1)	2.42 (2)	3.010 (2)	124 (2)
N4—H4*A*···O5′^iv^	0.89 (1)	2.58 (2)	3.273 (3)	136 (2)
N4—H4*B*···O1^v^	0.88 (1)	2.18 (1)	3.021 (2)	159 (2)

Symmetry codes: (i) −*x*+1, −*y*+1, −*z*+1; (ii) −*x*+1, −*y*+1, −*z*; (iii) *x*−1/2, *y*, −*z*+1/2; (iv) −*x*+3/2, −*y*+1, *z*−1/2; (v) *x*+1/2, *y*, −*z*+1/2.
